# Smoking-associated IgG4-positive cell infiltration in rheumatoid arthritis lung: an exploratory cross-sectional study

**DOI:** 10.3389/fimmu.2026.1873384

**Published:** 2026-08-14

**Authors:** Yoshika Tsuji, Tomohiro Koga, Nana Nakada, Atsushi Kawakami, Kazuko Yamamoto

**Affiliations:** 1 Department of Immunology and Rheumatology, Division of Advanced Preventive Medical Sciences, Nagasaki University Graduate School of Biomedical Sciences, Nagasaki, Japan; 2 Department of Respiratory Medicine, Nagasaki University Graduate School of Biomedical Sciences, Nagasaki, Japan; 3 DEJIMA Infectious Disease Research Alliance, Nagasaki University, Nagasaki, Japan; 4 Clinical Research Center, Nagasaki University Hospital, Nagasaki, Japan; 5 Research Career Support Office, Faculty of Biomedical Sciences, Nagasaki University, Nagasaki, Japan; 6 Department of Infectious Diseases, Respiratory Medicine, and Digestive Medicine (First Department of Internal Medicine), Faculty of Medicine, University of the Ryukyus, Ginowan, Japan; 7 Visiting Professor, Nagasaki University Graduate School of Biomedical Sciences, Nagasaki, Japan

**Keywords:** ACPA, bronchoalveolar lavage, cigarette smoking, flow cytometry, IgG4-related disease, interstitial lung disease, lung pathology, rheumatoid arthritis

## Abstract

**Introduction:**

Smoking is the first recognized modifiable risk factor for IgG4-related disease (IgG4-RD) and a driver of rheumatoid arthritis (RA)-associated interstitial lung disease, yet whether it is associated with IgG4-positive cell infiltration in RA lung tissue remains unclear.

**Methods:**

We analyzed 28 RA patients with abnormal chest CT findings undergoing bronchoalveolar lavage (BAL) and transbronchial lung biopsy; tissue IgG and IgG4 were quantified immunohistochemically. An exploratory IgG4/IgG ratio ≥10% defined IgG4-rich infiltration (only one patient met the conventional ≥40% pathology threshold). BAL and peripheral blood were phenotyped by flow cytometry. Comparisons used non-parametric tests, exploratory Firth penalized logistic regression to address quasi-complete separation, and exploratory subgroup and sensitivity analyses.

**Results:**

Six patients (21%) had IgG4/IgG ratio ≥10%; all were male ever-smokers with higher ACPA (343.1 vs 96.2 U/mL, p=0.015), although serum IgG4 did not differ (p=0.194). BAL fluid showed lower CD4/CD8 ratio, higher CD8, higher CD22+ B cells, and a trend toward higher Treg; peripheral blood T-helper subsets did not differ. IgG4-rich patients had more interstitial lung disease, more frequent reported probable UIP, higher emphysema scores, and lower baseline %FEV1.0. Tissue IgG4/IgG ratio correlated with ACPA and inversely with %FEV1.0. In an exploratory Firth analysis, male sex and log(ACPA) were associated with tissue IgG4 positivity; because all IgG4-rich cases were male ever-smokers, smoking and male sex could not be disentangled.

**Discussion:**

In this exploratory cohort, tissue IgG4-positive cell infiltration was observed only among male ever-smokers and was accompanied by a distinct BAL immune-cell profile without parallel peripheral-blood changes. Given the small sample size, the exploratory cutoff, and the unresolved confounding between sex and smoking, these findings should be regarded as hypothesis-generating and require validation in larger prospective studies.

## 1 Introduction

IgG4-related disease (IgG4-RD) is a systemic fibroinflammatory disorder characterized by elevated serum IgG4 concentrations and multi-organ infiltration by IgG4+ plasma cells, frequently accompanied by storiform fibrosis and obliterative phlebitis (1). The disease predominantly affects middle-aged and older men. Histologically, IgG4-RD is supported by dense lymphoplasmacytic infiltration, storiform fibrosis and/or obliterative phlebitis, together with increased IgG4+ plasma cells and an elevated IgG4/IgG ratio, although the precise thresholds vary by organ and by diagnostic framework (2, 3).

The lung is one of the more frequently involved organs in IgG4-RD and presents with heterogeneous findings including mediastinal lymphadenopathy, pulmonary nodules, bronchovascular thickening, pleural disease, and interstitial pneumonia (4, 5). Rheumatoid arthritis (RA) is also a disease in which pulmonary involvement, in particular interstitial lung disease (RA-ILD), is a major determinant of morbidity and mortality. Whether a distinct, local IgG4-positive cell response participates in RA lung pathology—rather than IgG4 being an incidental bystander of chronic airway injury—has not been directly examined. Smoking is a well-established risk factor for RA-ILD, with a threshold effect in which ≥30 pack-years confers an approximately sixfold increase in the risk of RA-ILD (6, 7).

Smoking has also emerged as the first modifiable risk factor for IgG4-RD itself. In a case–control study of 234 IgG4-RD cases and 1170 matched controls, current smokers had 1.79-fold higher odds of IgG4-RD (95% confidence interval [CI] 1.08 to 2.95), with the strongest signal among women and in patients with retroperitoneal fibrosis or normal serum IgG4 concentrations (8). Consistent with these findings, we previously reported in the Nagasaki Islands Study (NaIS; n=3240), a large health-checkup cohort in the general Japanese population, that smoking intensity is positively associated with serum IgG4 levels; similar findings have been reported in other Japanese cohorts (9, 10). These observations raise a specific question: whether smoking-related lung injury promotes a local IgG4-positive cell response within the lung, at the interface between IgG4-RD biology and RA-associated pulmonary autoimmunity.

However, whether these serum-level associations translate into tissue-level changes in the lung remains unclear. Ethical considerations preclude obtaining lung biopsy specimens from healthy individuals for research purposes, and available human data are restricted to clinically indicated biopsies. RA is a clinical population in which transbronchial lung biopsy (TBLB) is frequently undertaken to evaluate abnormal chest imaging and in which smoking-related pulmonary pathology is common; thus, it provides a useful model to investigate whether smoking is associated with IgG4-positive cell infiltration in lung tissue.

Mechanistically, IgG4-RD is characterized by expansion of circulating plasmablasts and a clonal expansion of CD4+ cytotoxic T lymphocytes, both in blood and in lesional tissue (11, 12); these features may be dissociated from serum IgG4 concentrations (12). In RA, by contrast, Klareskog and colleagues showed that cigarette smoking activates peptidylarginine deiminase (PAD) in the airways, drives citrullination of alveolar proteins and—in the context of the HLA-DR shared epitope—promotes anti-citrullinated protein antibody (ACPA) formation; importantly, citrullinated proteins were demonstrable by immunostaining in BAL cells from smokers but not non-smokers, identifying the lung as a site of initiation of RA-associated autoimmunity (13). Smoking thus has the capacity to promote local antigen exposure and antibody responses in the lung that are potentially relevant both to RA and to IgG4-RD. We therefore reasoned that the smoking-exposed RA lung is a biologically plausible site in which to seek IgG4-positive cell infiltration, directly linking the smoking–IgG4 association to the smoking–ACPA axis of RA-ILD pathogenesis.

In this context, we performed an integrated cross-sectional analysis in a consecutive cohort of RA patients undergoing BAL and TBLB for evaluation of abnormal chest CT findings. Our aim was not to diagnose IgG4-RD, but to determine whether IgG4-positive cell infiltration is present in RA lung tissue and whether it is associated with smoking-related pulmonary immune, structural, and functional changes. We hypothesized that smoking exposure is associated with pulmonary IgG4-positive cell infiltration in patients with RA; the study was not designed to diagnose IgG4-RD or to establish an RA–IgG4-RD overlap syndrome.

## 2 Materials and methods

### 2.1 Study design and participants

We conducted a single-center, retrospective, cross-sectional study at Nagasaki University Hospital. Eligible patients were consecutive adults with RA (diagnosed by a Japan College of Rheumatology-certified treating physician in accordance with the 2010 ACR/EULAR classification criteria) (14) who underwent BAL and TBLB for evaluation of abnormal chest CT findings—including reticulation and/or ground-glass opacity, nodular opacities, bronchiectasis, or other findings of uncertain etiology—between June 2019 and October 2021. Patients with available tissue specimens for immunohistochemical analysis were included in the final study population (n=28). The cohort therefore comprised a selected group of patients with RA who underwent clinically indicated bronchoscopy with BAL and TBLB, rather than an unselected RA population or a cohort restricted to RA-associated interstitial lung disease (RA-ILD); 14 of the 28 patients (50%) had CT-defined ILD. Because this was a retrospective study of patients evaluated in 2019–2021, the recently published ERS/EULAR recommendations for connective tissue disease–associated ILD were not applied prospectively as eligibility criteria. The study is reported in accordance with the STROBE statement (15); a completed checklist is provided as **Supplementary Material**.

### 2.2 Tissue immunohistochemistry and quantification of IgG4+ cells

Formalin-fixed, paraffin-embedded TBLB specimens were immunostained with a mouse anti-human IgG4 monoclonal antibody (clone HP6025, product code MC011; The Binding Site, Birmingham, UK; 1:2000) and a polyclonal rabbit anti-human IgG antibody (code A0423; Dako, Glostrup, Denmark; 1:50000), with diaminobenzidine (DAB) as chromogen and hematoxylin as counterstain. Staining was performed on deparaffinized sections with antigen retrieval using 0.1% trypsin in phosphate buffer (pH 7.6; 37 °C, 15 min), endogenous peroxidase blocking with 3% H2O2, and protein blocking (Blocking One, Nacalai Tesque, Kyoto, Japan); primary antibodies were applied overnight at 4 °C, followed by a peroxidase-labeled anti-mouse (for IgG4) or anti-rabbit (for IgG) IgG polyclonal secondary antibody (Nichirei, Tokyo, Japan), 3,3′-diaminobenzidine development, and Mayer’s hematoxylin counterstain, at a commercial pathology laboratory (Kyodo Byori, Kobe, Japan). For each patient, paired high-power (×400) images of the same field stained for IgG and for IgG4 were captured digitally (1920×1440 pixels). Candidate positive cells were extracted in the HSV color space as DAB-positive pixels and segmented into individual cells by connected-component analysis followed by watershed segmentation, using Python with the scikit-image and Pillow libraries. Automated counts were then manually verified under microscopy, and a candidate was retained as a positive cell only when all three of the following criteria were met: (i) a clearly visible nuclear-negative area, (ii) homogeneous DAB-positive cytoplasmic staining, and (iii) recognition as an isolated, independent cell. The IgG4+ cells enumerated in this way displayed morphological features consistent with plasma cells (eccentric nuclei and abundant cytoplasm) on the IgG4-stained sections; however, IgG4 was applied as a single chromogenic immunostain, and colocalization with dedicated plasma-cell markers (CD38, CD138, or IRF4) by dual or multiplex immunostaining was not performed in this retrospective cohort. Diffuse interstitial staining, alveolar intraluminal protein-like material, dust-laden macrophages with fine granular cytoplasmic pigment (carbon-laden macrophages), the halo around charcoal aggregates, and edge artifacts were excluded. The final per-field count is the visually verified value rather than the raw automated count. We report both IgG4+ cell counts per high-power field (HPF) and the IgG4/IgG cell ratio. All fields were assessed independently by two observers blinded to the clinical, smoking, and radiological data, and discrepancies were resolved by consensus.

Field selection followed a hot-spot approach in which the most densely IgG-stained area on the IgG-stained slide was localized and the corresponding field was then captured on the IgG4-stained slide, in line with the approach commonly used for IgG4-RD pathology assessment (16). A representative IgG4-rich case is illustrated in **Supplementary Figure 1**.

In our cohort, only one of 28 patients (3.6%) met the IgG4-RD pathology threshold of IgG4/IgG ≥40%. Because this would yield an uninformatively small “IgG4-positive” group, we adopted a lower, exploratory threshold of IgG4/IgG ratio ≥10% to define IgG4-rich infiltration for the principal between-group comparisons. We emphasize that this ≥10% threshold is used as an exploratory low-threshold definition of IgG4-rich infiltration and not as a diagnostic threshold for IgG4-RD. To address concerns about threshold dependence, we performed sensitivity analyses at thresholds of 5%, 10%, 20%, and 40% (**Supplementary Table 1**), Spearman analyses treating the IgG4/IgG ratio and IgG4+ cells/HPF as continuous variables, and analyses of IgG4+ cells/HPF as a continuous count.

### 2.3 Flow cytometry of peripheral blood and BAL fluid

Peripheral blood was collected into BD Vacutainer CPT tubes (BD Biosciences, San Jose, CA, USA), and peripheral blood mononuclear cells (PBMCs) were isolated by density-gradient centrifugation according to the manufacturer’s instructions. PBMCs and BAL fluid cells were stained for 30 min at 4 °C with antibodies against CD3 (clone SK7; BioLegend, San Diego, CA, USA), CD4 (SK3; BioLegend), CD45RA (HI100; BioLegend), CCR7 (G043H7; BioLegend), CCR6 (G034E3; BioLegend), CXCR3 (G025H7; BioLegend), CD25 (BC96; BioLegend), and CD127 (A019D5; BioLegend); remaining antibodies were also obtained from BioLegend. Dead cells were excluded using 7-AAD Viability Staining Solution (BioLegend). Data were acquired on a BD FACSLyric (BD Biosciences) and analyzed using FlowJo v10.8.2 (Tree Star, Ashland, OR, USA). Regulatory T cells (Treg) were defined as CD127−CD25+ within CD4+ T cells; Th1 as CCR6−CXCR3+, Th2 as CCR6−CXCR3−, Th17 as CCR6+CXCR3+, and CCR6+CXCR3− Th cells were enumerated as separate Th subsets. The gating strategy for BAL fluid and peripheral blood flow cytometry is provided in **Supplementary Figure 3**.

### 2.4 Clinical, radiological, and physiological data

For each participant we extracted age, sex, smoking history (status, pack-years, years since cessation), body mass index, RA disease duration, comorbidities, vaccination history, serological data (rheumatoid factor [RF], ACPA, ESR, C-reactive protein [CRP], IgG4, IgG, complement components, IgE, CH50), RA disease activity measures (patient and physician VAS, 28-joint tender [TJC] and swollen [SJC] counts, DAS28-CRP, DAS28-ESR, SDAI, and CDAI with corresponding categories), and treatment exposures. The overall fibrotic HRCT pattern was categorized as UIP, probable UIP, indeterminate for UIP, or an alternative diagnosis (corresponding to the ATS/ERS/JRS/ALAT categories), and individual CT features (interstitial lung disease, ground-glass opacity, reticulation, traction bronchiectasis, honeycombing, centrilobular nodules, mediastinal lymphadenopathy, bronchial wall thickening, mucoid impaction, centrilobular emphysema score 0–5, paraseptal emphysema score 0–2, and modified Reiff score) were recorded separately. All radiological variables were abstracted from the clinical radiology reports issued at the time of TBLB and were not centrally re-evaluated in a blinded fashion for the present study. Pulmonary function tests (VC, %VC, FEV1.0, %FEV1.0) were recorded at baseline.

### 2.5 Statistical analysis

Continuous variables are summarized as median [first quartile, third quartile] and categorical variables as n (%). Between-group comparisons used the Mann–Whitney U test for continuous and the Fisher exact test for categorical variables. Spearman rank correlation was used for continuous-by-continuous associations between tissue IgG4 measures (IgG4/IgG ratio and IgG4+ cells/HPF) and BAL flow-cytometric, serological, and physiological indices.

Sensitivity analyses examined the robustness of the principal between-group comparisons at four thresholds for tissue IgG4 positivity (≥5%, ≥10%, ≥20%, ≥40%); the ≥40% threshold was retained for completeness despite yielding only n=1 (**Supplementary Table 1**). Subgroup analyses repeated the IgG4-positive vs IgG4-negative comparison after restricting the cohort to (i) men only (n=9), (ii) ever-smokers only (n=13), and (iii) patients with CT-defined ILD (n=14), to evaluate whether differences in BAL immune profile, ACPA, and %FEV1.0 persisted within strata defined by sex, smoking, or lung disease status (**Supplementary Tables 2**-**4**).

Logistic-regression analyses of tissue IgG4 positivity were exploratory only. Because tissue IgG4 positivity was confined to men who were all ever-smokers, conventional maximum-likelihood logistic regression was precluded by quasi-complete separation. We therefore used Firth’s penalized maximum-likelihood logistic regression (17), which yields finite estimates and CIs under separation (18). An exploratory four-variable Firth model (sex, ever-smoker, log[ACPA + 0.1], UIP pattern) was fitted, but is interpreted with caution given the small effective sample size (n=28, six events) and is reported as exploratory; primary inference relies on the descriptive between-group comparisons, sensitivity and subgroup analyses, and continuous correlations. Because the regression was exploratory and intended to estimate associations rather than to develop or validate a predictive model, no training–validation split or external validation cohort was used; given the small number of outcome events (six), the number of covariates was deliberately limited to reduce overfitting, and conventional *post-hoc* multiple-comparison testing is not routinely required following logistic regression.

Analyses were performed using Python 3.10 with the firthlogist, scipy (19), statsmodels, and scikit-image (20) packages. No adjustment was made for multiple testing, given the hypothesis-generating nature of the study; false-discovery-rate-adjusted q-values for the principal panel of comparisons are provided in the **Supplementary Material**. Statistical significance was set at two-sided p<0.05.

## 3 Results

### 3.1 Cohort characteristics and distribution of tissue IgG4

**Figure 1:**
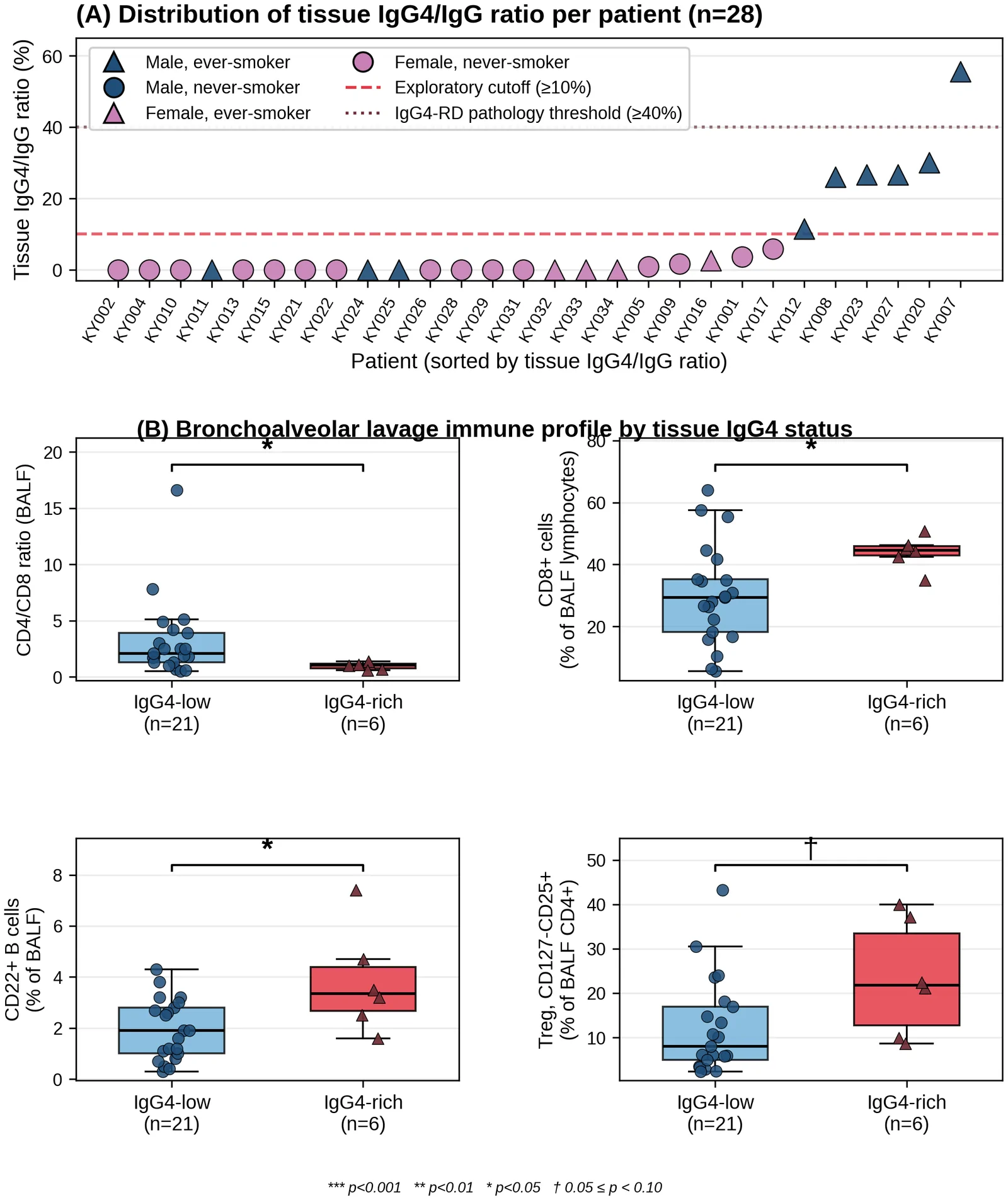
Distribution of tissue IgG4/IgG ratio and bronchoalveolar lavage (BAL) immune profile according to IgG4 status. **(A)** Distribution of tissue IgG4/IgG ratio across the cohort (n=28). Each marker represents one patient; sex is encoded by color (blue = male; pink = female) and smoking status by shape (triangle = ever-smoker; circle = never-smoker). The dashed red line indicates the exploratory cutoff (≥10%) used for principal between-group comparisons; the dotted line indicates the IgG4-RD pathology threshold (≥40%). Only one patient met the IgG4-RD pathology threshold. **(B)** BAL fluid immune profile by tissue IgG4 status. Box-and-individual-data plots for BAL CD4/CD8 ratio, CD8+ cells (% of BAL lymphocytes), CD22+ B cells (% of BAL fluid), and Treg (CD127−CD25+ within CD4+ T cells). Triangles denote IgG4-rich (ratio ≥10%) and circles denote IgG4-low patients. Statistical significance is indicated as ***p<0.001, **p<0.01, *p<0.05, and † 0.05 ≤ p < 0.10 (Mann–Whitney U test).

Of the 28 RA patients who underwent BAL and TBLB for abnormal chest CT findings, 17 (61%) had a tissue IgG4/IgG ratio of 0%, six (21%) had a ratio ≥10% (IgG4-rich group), and one (3.6%) met the IgG4-RD pathology threshold of ≥40% (**Figure 1A**). The median IgG4+ cell count was 0/HPF (range 0–80), and only four (14%) patients had ≥10 IgG4+ cells/HPF. Clinical, serological, RA-activity, and treatment characteristics are shown in **Table 1**. All six IgG4-rich patients were men (vs 3/22 in the IgG4-low group, p&lt;0.001), all were ever-smokers (vs 7/22, p=0.005), and they had higher ACPA concentrations (343.1 [303.3, 461.8] vs 96.2 [27.7, 239.6] U/mL, p=0.015). Median pack-years among ever-smokers did not differ between groups (900 [780, 975] vs 420 [158, 1350], p=0.667). Serum IgG4 levels did not differ significantly (83.7 [65.5, 98.7] vs 54.3 [30.8, 74.6] mg/dL, p=0.194). RF, complement, IgE, BMI, RA disease duration, and comorbidities did not differ between groups.

**Table 1:** Clinical and serological characteristics, RA disease activity and treatment exposures, stratified by tissue IgG4/IgG ratio.

Variable	IgG4-low (ratio <10%, n=22)	IgG4-rich (ratio ≥10%, n=6)	p-value
Age, years	68 [61, 74]	68 [65, 71]	1.000
Male sex, n (%)	3 (13.6)	6 (100.0)	<0.001
Ever-smoker, n (%)	7 (31.8)	6 (100.0)	0.005
Pack-years (ever-smokers only)	420 [158, 1350]	900 [780, 975]	0.667
Years since smoking cessation	52 [41, 67]	57 [57, 65]	0.764
Tissue IgG4/IgG ratio (%)	0.0 [0.0, 0.0]	26.7 [26.2, 29.2]	<0.001
Tissue IgG4^+^ cells/HPF	0 [0, 1.5]	12 [4, 53]	<0.001
Serum IgG4 (mg/dL)	54.3 [30.8, 74.6]	83.7 [65.5, 98.7]	0.194
CRP (mg/dL)	0.77 [0.23, 2.40]	0.78 [0.57, 2.70]	0.484
ESR 2h (mm/hr)	73 [47, 92]	76 [49, 82]	0.838
ACPA (U/mL)	96.2 [27.7, 239.6]	343.1 [303.3, 461.8]	0.015
Rheumatoid factor (IU/mL)	76.4 [47.5, 201.9]	50.4 [31.6, 185.9]	0.932
C3 (mg/dL)	147 [120, 157]	140 [130, 162]	1.000
C4 (mg/dL)	28 [18, 34]	19 [18, 22]	0.262
IgE (IU/mL)	46.7 [18.9, 418.8]	316.8 [27.0, 598.0]	0.385
BMI (kg/m²)	20.8 [19.4, 22.8]	22.9 [21.6, 23.6]	0.123
RA duration (years)	4.5 [3.0, 10.5]	3.0 [2.3, 8.3]	0.651
— RA disease activity —			
Patient global VAS (mm)	19.50 [6.62, 35.50]	37.00 [14.75, 45.75]	0.370
TJC (/28)	2.00 [0.25, 4.75]	3.00 [2.00, 7.00]	0.313
SJC (/28)	1.50 [0.00, 4.25]	2.50 [0.50, 6.75]	0.584
Patient VAS	19.50 [6.62, 35.50]	37.00 [14.75, 45.75]	0.370
Physician VAS	19.50 [6.62, 35.50]	37.00 [14.75, 45.75]	0.370
DAS28-CRP	3.39 [1.93, 4.08]	3.80 [3.44, 4.18]	0.283
DAS28-ESR	4.57 [3.53, 5.21]	4.86 [4.38, 5.13]	0.476
SDAI	10.93 [2.83, 17.71]	14.77 [10.68, 20.84]	0.427
CDAI	9.00 [2.75, 16.35]	11.90 [9.85, 19.20]	0.395
ESR 1h (mm/hr)	73.00 [47.00, 92.00]	75.50 [48.50, 82.25]	0.838
CH50 (U/mL)	12.00 [12.00, 12.00]	12.00 [12.00, 12.00]	0.663
— Medication use —			
NSAID use	12/22 (54.5%)	5/6 (83.3%)	0.355
Sulfasalazine use	3/22 (13.6%)	1/6 (16.7%)	1.000
csDMARD use	4/22 (18.2%)	1/6 (16.7%)	1.000
Methotrexate use	10/22 (45.5%)	1/6 (16.7%)	0.355
Glucocorticoid use	5/22 (22.7%)	2/6 (33.3%)	0.622
Other immunosuppressant use	3/22 (13.6%)	0/6 (0.0%)	1.000
JAK inhibitor use	0/22 (0.0%)	0/6 (0.0%)	1.000
Biologic/JAK inhibitor use	4/22 (18.2%)	0/6 (0.0%)	0.549

Data are median [first quartile, third quartile] for continuous variables and n (%) for categorical variables. p-values: Mann–Whitney U test for continuous, Fisher exact test for categorical variables. Pack-years and years since cessation were analysed among ever-smokers only. ACPA and IgE values reported as below or above assay limits were retained at the limit value for rank-based tests. Yellow shading: p<0.05; orange shading: 0.05 ≤ p <0.10.

ACPA, anti-citrullinated peptide antibody; BMI, body mass index; CDAI, Clinical Disease Activity Index; CRP, C-reactive protein; csDMARD, conventional synthetic disease-modifying antirheumatic drug; DAS28, 28-joint disease activity score; ESR, erythrocyte sedimentation rate; HPF, high-power field; JAK, Janus kinase; NSAID, non-steroidal anti-inflammatory drug; RA, rheumatoid arthritis; SDAI, Simplified Disease Activity Index; SJC, swollen joint count; TJC, tender joint count; VAS, visual analogue scale.

### 3.2 RA disease activity and treatment

Measures of RA disease activity—VAS, Pt./Dr.VAS, TJC, SJC, DAS28-CRP, DAS28-ESR, SDAI, CDAI, including the corresponding categorical classifications—were similar between groups (**Table 1**). Use of NSAIDs, sulfasalazine, conventional synthetic DMARDs, MTX, glucocorticoids, other immunosuppressants, JAK inhibitors, and biological DMARDs did not differ. Tissue IgG4-rich status was therefore not explained by greater joint inflammation or by heavier immunomodulatory treatment.

### 3.3 BAL fluid flow cytometry

In BAL fluid (**Table 2**; **Figure 1B**), IgG4-rich patients had a lower CD4/CD8 ratio (1.05 [0.77, 1.18] vs 2.10 [1.30, 3.90], p=0.017), lower CD4 (p=0.036), higher CD8 (p=0.022), and higher CD22+ B cells (3.35 [2.67, 4.40] vs 1.90 [1.00, 2.80] %, p=0.029). Treg within BAL CD4+ T cells were higher, with borderline statistical significance (21.8 [12.7, 33.4] vs 8.0 [5.0, 17.0] %, p=0.057). BAL Th1, Th2, Th17, CCR6+CXCR3− Th, CD14+CD16− macrophages, CD86+CD14+ activated macrophages, and CD66b+ neutrophil subsets did not differ.

**Table 2:** Bronchoalveolar lavage fluid cellular and flow-cytometric findings, stratified by tissue IgG4/IgG ratio.

Variable	n (IgG4-rich)	IgG4-rich [Q1, Q3]	n (IgG4-low)	IgG4-low [Q1, Q3]	p-value
BAL fluid volume (mL)	6	20.00 [20.00, 20.00]	21	20.00 [19.00, 20.00]	0.639
BAL WBC (×10^6^/mL)	6	0.25 [0.21, 0.33]	21	0.21 [0.15, 0.48]	0.798
Macrophages (%)	6	90.00 [87.25, 92.00]	21	71.00 [55.00, 92.00]	0.129
Lymphocytes (%)	6	6.00 [5.25, 9.00]	21	16.00 [5.00, 29.00]	0.209
Neutrophils (%)	6	2.00 [1.25, 3.50]	21	5.00 [1.00, 13.00]	0.411
Eosinophils (%)	6	1.00 [0.25, 1.75]	21	0.00 [0.00, 1.00]	0.421
Basophils (%)	6	0.00 [0.00, 0.00]	21	0.00 [0.00, 0.00]	0.075
Plasma cells (%)	6	0.00 [0.00, 0.00]	21	0.00 [0.00, 0.00]	1.000
CD3^+^ (%)	6	87.75 [83.70, 90.08]	21	92.00 [89.20, 95.00]	0.085
CD4^+^ (%)	6	45.20 [34.45, 48.23]	21	62.60 [45.60, 69.10]	0.036
CD8^+^ (%)	6	44.65 [42.88, 45.97]	21	29.40 [18.20, 35.20]	0.022
CD4/CD8 ratio	6	1.05 [0.77, 1.18]	21	2.10 [1.30, 3.90]	0.017
CD22^+^ B cells (%)	6	3.35 [2.67, 4.40]	21	1.90 [1.00, 2.80]	0.029
CD16^+^ (%)	6	3.10 [2.78, 6.88]	21	2.60 [1.80, 6.40]	0.448
CD56^+^ (%)	6	7.10 [4.15, 10.28]	21	4.00 [3.40, 7.50]	0.366
Treg, CD127^-^CD25^+^ (% of BALF CD4^+^)	6	21.80 [12.74, 33.42]	21	7.98 [4.98, 17.00]	0.057
Th1, CCR6^-^CXCR3^+^ (%)	6	41.90 [33.62, 55.35]	21	32.10 [10.70, 43.60]	0.175
Th2, CCR6^-^CXCR3^-^ (%)	6	29.00 [16.25, 46.62]	21	35.40 [12.40, 61.30]	0.932
Th17, CCR6^+^CXCR3^+^ (%)	6	10.79 [6.45, 18.45]	21	11.90 [5.45, 24.20]	0.884
Th, CCR6^+^CXCR3^-^ (%)	6	6.21 [4.50, 8.21]	21	10.90 [4.46, 16.10]	0.512
Macrophages (CD14^+^CD16^-^, %)	5	21.70 [11.90, 45.10]	20	25.00 [10.43, 38.73]	0.921
Activated macrophages (CD86^+^CD14^+^, %)	5	78.90 [75.50, 82.10]	20	82.65 [71.22, 91.12]	0.812
Neutrophils (CD66b^+^CD16^+^, %)	2	18.20 [16.35, 20.05]	4	17.75 [11.53, 29.42]	1.000
Neutrophils (CD66b^+^CD206^-^, %)	5	16.50 [14.00, 23.10]	18	32.95 [16.43, 62.45]	0.150

Values are median [first quartile, third quartile]. Mann–Whitney U test. Yellow shading: p<0.05; orange shading: 0.05 ≤ p <0.10. Patients with missing values for a given variable were excluded from that comparison. Peripheral blood flow-cytometric data are reported in **Supplementary Table 6**.

BAL, bronchoalveolar lavage; CCR, C-C chemokine receptor; CXCR, C-X-C chemokine receptor; Th, T helper cell; Treg, regulatory T cell.

### 3.4 Peripheral blood flow cytometry

In peripheral blood (**Supplementary Table 6**), no significant differences were observed in Treg, Th1, Th2, Th17, or CCR6+CXCR3− Th percentages, nor in total leukocyte, neutrophil, lymphocyte, monocyte, eosinophil, or basophil counts. Peripheral blood CD66b+CD206− neutrophils were numerically higher but not statistically significant in the IgG4-rich group (83.6 [75.6, 85.9] vs 53.1 [39.3, 73.5] %, p=0.104). The contrast between significant BAL differences and absent peripheral blood differences is the key observation supporting a lung-local process.

### 3.5 Chest CT findings, pulmonary function, and continuous correlations

**Figure 2:**
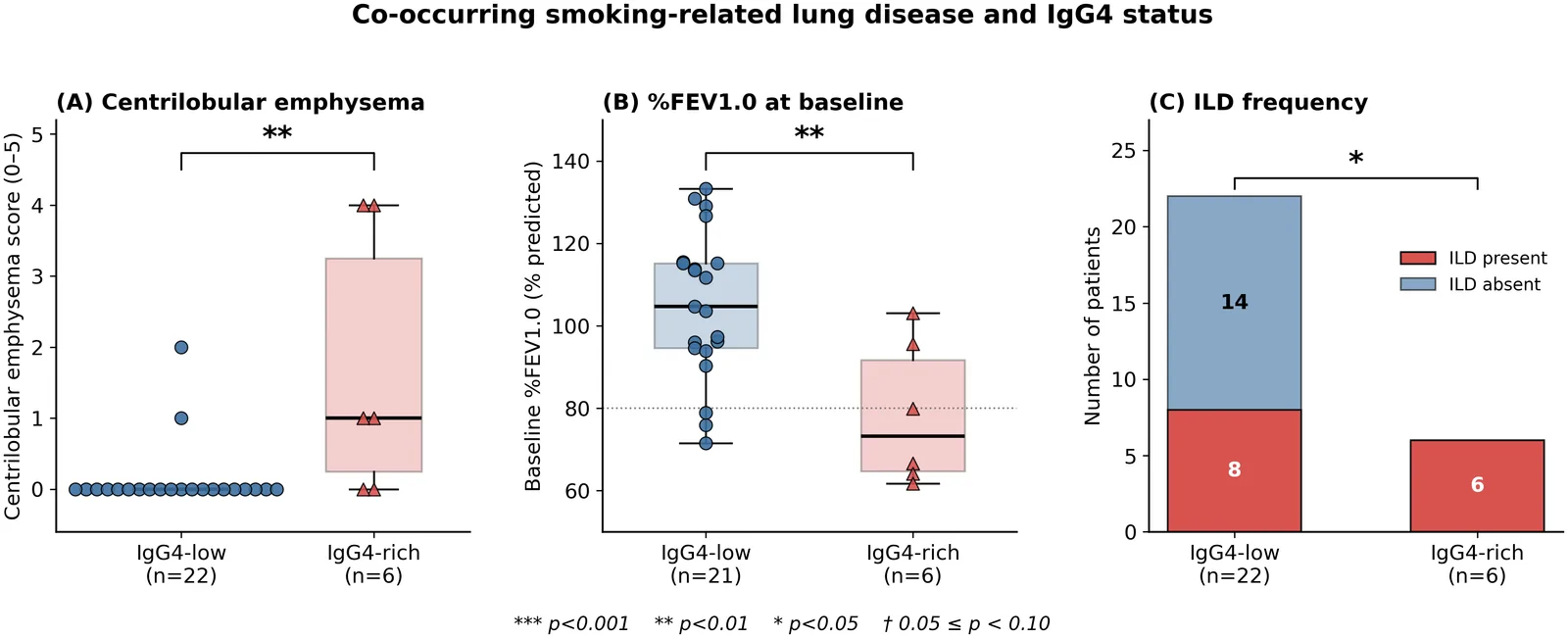
Co-occurring smoking-related lung disease and IgG4 status. **(A)** Centrilobular emphysema score (0–5) by IgG4 status. **(B)** Baseline %FEV1.0 by IgG4 status; the dotted gray line indicates the conventional lower limit of normal at 80%. **(C)** Stacked bar of ILD frequency: ILD was present in all six IgG4-rich patients but in only 8 of 22 IgG4-low patients. Statistical significance is indicated as ***p<0.001, **p<0.01, *p<0.05, and † 0.05 ≤ p < 0.10 [Mann–Whitney U test for **(A)** and **(B)**; Fisher exact test for **(C)**].

ILD was present in all six IgG4-rich patients (100%) but in only 8 of 22 IgG4-low patients (36%, p=0.016) (**Table 3**, **Figure 2C**). A higher frequency of reported probable UIP pattern was observed in the IgG4-rich group (2/6 vs 0/22, p=0.040). Centrilobular emphysema score (median 1.0 vs 0.0, p=0.003; **Figure 2A**) and paraseptal emphysema score (0.5 vs 0.0, p=0.005) were higher in IgG4-rich patients. Baseline %FEV1.0 was lower in the IgG4-rich group (73.3 [64.7, 91.7] vs 104.7 [94.6, 115.2], p=0.009; **Figure 2B**), indicating an obstructive ventilatory pattern.

**Table 3:** Chest CT findings and baseline pulmonary function, stratified by tissue IgG4/IgG ratio.

Variable	IgG4-low (n=22)	IgG4-rich (n=6)	p-value
Abnormal CT findings	22/22 (100.0%)	6/6 (100.0%)	1.000
Interstitial lung disease	8/22 (36.4%)	6/6 (100.0%)	0.016
UIP pattern	2/22 (9.1%)	1/6 (16.7%)	0.530
Probable UIP	0/22 (0.0%)	2/6 (33.3%)	0.040
Indeterminate for UIP	2/22 (9.1%)	2/6 (33.3%)	0.191
Centrilobular nodules	10/22 (45.5%)	2/6 (33.3%)	0.673
Mediastinal lymphadenopathy	2/22 (9.1%)	2/6 (33.3%)	0.191
Bronchial wall thickening	11/22 (50.0%)	4/6 (66.7%)	0.655
Mucoid impaction	9/22 (40.9%)	0/6 (0.0%)	0.136
Bronchiectasis	11/22 (50.0%)	3/6 (50.0%)	1.000
Centrilobular emphysema score (0–5)	0.00 [0.00, 0.00]	1.00 [0.25, 3.25]	0.003
Paraseptal emphysema score (0–2)	0.00 [0.00, 0.00]	0.50 [0.00, 1.75]	0.005
Modified Reiff score	0.75 [0.00, 3.00]	0.50 [0.00, 1.00]	0.250
— Pulmonary function (baseline) —			
VC (L)	2.77 [2.51, 3.04]	3.60 [2.93, 3.95]	0.057
VC, % predicted	118.40 [103.60, 121.60]	99.45 [90.78, 114.50]	0.115
FEV_1_ (L)	2.20 [1.66, 2.28]	2.23 [1.73, 2.50]	0.683
FEV_1_, % predicted	104.70 [94.60, 115.20]	73.25 [64.72, 91.67]	0.009

Binary CT findings are expressed as n with the finding/total n assessed (%) and were compared with the Fisher exact test. Ordinal emphysema scores (centrilobular 0–5; paraseptal 0–2) and the modified Reiff score, together with spirometric values, are expressed as median [Q1, Q3] and were compared with the Mann–Whitney U test.

CT, computed tomography; FEV_1_, forced expiratory volume in 1 second; ILD, interstitial lung disease; UIP, usual interstitial pneumonia; VC, vital capacity. Yellow shading, p<0.05; orange shading, 0.05<=p<0.10.

As continuous variables, tissue IgG4/IgG ratio correlated positively with ACPA (Spearman ρ=0.50, p=0.007) and inversely with baseline %FEV1.0 (ρ=−0.39, p=0.044). IgG4+ cells/HPF likewise correlated positively with ACPA (ρ=0.44, p=0.018) and showed a non-significant inverse correlation with %FEV1.0 (ρ=−0.33, p=0.090). Correlations of tissue IgG4 measures with BAL CD4/CD8 ratio, CD22+, CD8, and Treg were in the expected directions but did not reach statistical significance, reflecting the bimodal distribution (most IgG4-low patients clustered at 0%). The full set of Spearman correlations is provided in **Supplementary Table 5**.

### 3.6 Sensitivity analyses across thresholds

Across thresholds of ≥5%, ≥10%, and ≥20%, IgG4-rich status was significantly associated with male sex (p=0.001, <0.001, and 0.001 respectively), ever-smoking (p=0.029, 0.005, and 0.013), and higher ACPA (p=0.038, 0.015, and 0.058). At the IgG4-RD pathology threshold of ≥40%, only one patient was reclassified as positive and no comparison was statistically meaningful. The ≥10% threshold therefore offered the most stable signal across sex, smoking, and ACPA in this cohort, supporting its use as an exploratory operational definition (without implying diagnostic validity for IgG4-RD).

### 3.7 Subgroup analyses

To examine whether the principal differences between IgG4-rich and IgG4-low groups were driven by the male/ever-smoker composition of the IgG4-rich group or by the high prevalence of ILD, we repeated comparisons in three pre-defined subgroups.

Among the nine men in the cohort (six IgG4-rich, three IgG4-low; **Supplementary Table 2**), IgG4+ cell counts (12 [4, 53] vs 0 [0, 0]/HPF, p=0.025), ACPA (343.1 vs 0.6 U/mL, p=0.027), and baseline %FEV1.0 (73.3 vs 103.6, p=0.048) remained significantly different between groups.

Among the 13 ever-smokers (six IgG4-rich, seven IgG4-low; **Supplementary Table 3**), IgG4+ cell counts remained markedly different (12 vs 0/HPF, p=0.002), as did baseline %FEV1.0 (73.3 vs 103.6, p=0.022); ACPA (p=0.052), BAL CD4/CD8 ratio (p=0.099), male sex (p=0.070), and ILD prevalence (p=0.070) approached significance.

Among the 14 patients with CT-defined ILD (six IgG4-rich, eight IgG4-low; **Supplementary Table 4**), IgG4+ cell counts (12 vs 0/HPF, p=0.019) and %FEV1.0 (73.3 vs 108.6, p=0.013) remained significantly different, and BAL CD22 was higher at borderline significance (p=0.053). Within ILD, IgG4-rich status was still associated with male sex (p=0.010) and ever-smoking (p=0.031).

Together, these subgroup analyses indicate that the BAL immune profile, the ACPA elevation, and the obstructive pulmonary function pattern of the IgG4-rich group are not solely a consequence of being male, of being a smoker, or of having ILD.

### 3.8 Exploratory Firth penalized logistic regression

In univariable Firth regression for tissue IgG4 positivity, male sex (OR 72.4, 95% CI 6.33 to >1000; p<0.001), ever-smoking (OR 26.9, 95% CI 2.60 to >1000; p=0.003), and log(ACPA) (OR 2.87, 95% CI 1.04 to 15.90; p=0.036) were associated with IgG4 positivity, whereas UIP pattern (OR 2.24; p=0.492) and age (OR 1.00; p=0.939) were not (**Table 4**). The pre-specified four-variable multivariable Firth model (n=28, six events; events-per-variable 1.5) is exploratory; in this model, male sex (OR 37.2, 95% CI 1.91 to >1000; p=0.012) and log(ACPA) (OR 1.88, 95% CI 1.03 to 84.0; p=0.033) remained associated with tissue IgG4 positivity, whereas ever-smoking (p=0.684) and UIP (p=0.617) did not. We emphasize that all IgG4-rich patients were men and ever-smokers, so the independent contribution of smoking cannot be statistically separated from that of male sex; the Firth point estimates and CIs should be regarded as a sensitivity check on the descriptive analyses rather than as definitive multivariable inference.

**Table 4:** Firth penalised logistic regression for tissue IgG4 positivity (IgG4/IgG ratio ≥10%) as outcome.

Variable	n	Univariable OR [95% CI]	Univariable p	Multivariable*
Male sex (1/0)	28	72.43 [6.33, 10304.47]	0.000	OR 37.15 [1.91, 7575.25]; p=0.012
Ever-smoker (1/0)	28	26.87 [2.60, 3682.07]	0.003	OR 1.88 [0.01, 380.36]; p=0.684
log(ACPA + 0.1)	28	2.87 [1.04, 15.90]	0.036	OR 1.88 [1.03, 83.99]; p=0.033
UIP pattern (1/0)	28	2.24 [0.18, 20.83]	0.492	OR 2.79 [0.01, 47728827.12]; p=0.617
Age (years)	28	1.00 [0.92, 1.11]	0.939	—

*The four-variable multivariable model (sex + ever-smoker + log[ACPA] + UIP, n=28, six events) is interpreted as exploratory only because of the small effective sample size and the quasi-complete separation between male sex/smoking and IgG4 positivity. Firth penalised maximum-likelihood logistic regression yields finite estimates and CIs under separation. Age was examined only in the univariable analysis.

ACPA, anti-citrullinated peptide antibody; CI, confidence interval; OR, odds ratio; UIP, usual interstitial pneumonia.

## 4 Discussion

In this single-center cross-sectional study of RA patients undergoing BAL and TBLB for evaluation of abnormal chest CT findings, an exploratory tissue IgG4/IgG threshold of ≥10% (not the IgG4-RD diagnostic threshold of ≥40%) identified six (21%) patients with IgG4-positive cell infiltration. This subgroup was confined to male ever-smokers with higher ACPA, exhibited a lung-local immune profile (lower BAL CD4/CD8 ratio, expanded CD22+ B cells, with a trend toward higher Treg) without parallel changes in peripheral blood, and showed co-occurring smoking-related lung disease with reduced %FEV1.0. RA disease activity, treatment exposure, RF, and serum IgG4 did not differ between groups. The proposed conceptual relationships among smoking-related lung injury, lung-local immune shifts, ACPA, and tissue IgG4-rich infiltration are summarized in **Supplementary Figure 2**. To our knowledge, this is the first integrated tissue–BAL–blood–imaging–physiology characterization of IgG4-positive lung infiltration in a clinically selected RA cohort undergoing TBLB.

Because comparisons involving tissue IgG4 could be confounded by RA disease activity or by differential immunomodulatory therapy, we examined a comprehensive panel of activity indices, serological activity markers, and treatment exposures. None differed significantly between IgG4-rich and IgG4-low groups. Within ILD-positive, ever-smoker, and male subgroups, the principal differences (IgG4+ cells/HPF, ACPA, %FEV1.0) were preserved. Together, these findings argue that tissue IgG4-rich infiltration in RA patients undergoing TBLB is not simply a marker of more active synovitis or of heavier systemic treatment.

Classical IgG4-RD is accompanied by systemic immunological changes: circulating plasmablasts are expanded in active disease even when serum IgG4 is normal (12, 21), and clonally expanded CD4+ cytotoxic T lymphocytes are present in both blood and lesional tissue (11, 22). In our cohort, peripheral blood Treg, Th1, Th2, and Th17 percentages were comparable between IgG4-rich and IgG4-low patients, whereas BAL fluid showed a coherent set of differences (lower CD4/CD8 ratio, expanded CD22+ B cells, with a trend toward higher Treg). The contrast suggests that the IgG4-rich infiltration identified here appears predominantly local, and is therefore phenotypically distinct from classical systemic IgG4-RD. This observation is also consistent with the report that the association of smoking with IgG4-RD is strongest in subgroups with normal serum IgG4 (8), in which local rather than systemic features may dominate.

The co-occurrence within BAL fluid of a reduced CD4/CD8 ratio and an expanded CD22+ B-cell population may appear counterintuitive but is compatible with a compartmentalized local lymphoid response. The lower CD4/CD8 ratio was driven principally by an increase in CD8+ T cells, a well-recognized feature of the smoker’s and chronically injured lower airway, whereas the higher CD22+ B-cell fraction reflects the B-lineage compartment; the two are not mutually exclusive. Chronic airway injury and smoking promote inducible bronchus-associated lymphoid tissue (iBALT) and ectopic lymphoid aggregates, which characteristically contain both CD8+ T cells and B cells and provide a local niche for antibody-producing plasma cells. A CD8-skewed T-cell compartment coexisting with local B-cell accumulation is therefore consistent with a lung-restricted lymphoid reaction in which local B-cell help and antigen exposure, rather than a systemic T-helper shift, support IgG4-positive cell infiltration. These associations are interpreted cautiously given the small sample size and the descriptive nature of the flow-cytometric comparisons.

All six IgG4-rich patients were ever-smokers, and tissue IgG4/IgG ratio correlated positively with ACPA on continuous analysis (ρ=0.50, p=0.007). In Firth multivariable analysis (interpreted as exploratory only), log(ACPA) remained associated with tissue IgG4 positivity after adjustment for sex, smoking, and UIP pattern. This model should be interpreted as an exploratory sensitivity analysis rather than as evidence of an independent smoking effect, because all IgG4-positive cases were male ever-smokers and the contributions of smoking and male sex cannot be separated. Smoking is known to activate PAD in the airways, to drive citrullination of alveolar proteins in the lung, and—in the context of the HLA-DR shared epitope—to promote ACPA formation; critically, citrullinated proteins are demonstrable by immunostaining in BAL cells from smokers but not non-smokers (13). The present associations are compatible with a model in which smoking-related lung damage may provide a permissive microenvironment for local IgG4-positive cell accumulation. The absence of a significant difference in pack-years between groups (900 vs 420) and the lack of a continuous correlation between pack-years and tissue IgG4 measures suggest that, beyond a threshold of cumulative exposure, the relevant factor may be the presence versus absence of smoking-related lung remodeling rather than a linear function of pack-years; this parallels the threshold effect reported for RA-ILD, in which risk rises sharply at ≥30 pack-years (6). Causality cannot be inferred from cross-sectional data, however, and these statements remain hypothesis-generating.

Radiological and functional features in the IgG4-rich group—universal ILD, more frequent reported probable UIP pattern, higher emphysema scores, and reduced %FEV1.0—indicate that IgG4-rich infiltration in this cohort co-occurs with established smoking-related structural and functional changes. We cannot determine from these data whether tissue IgG4-rich infiltration represents a preclinical IgG4-RD process, a non-specific local cell response to chronic airway injury, or a candidate RA-lung phenotype. Only one of 28 patients met the IgG4-RD pathology threshold of IgG4/IgG ≥40%, indicating that IgG4-rich infiltration in this cohort does not in itself amount to a diagnosis of IgG4-RD. Accordingly, our findings should not be interpreted as indicating IgG4-related disease or a conventional IgG4-related histopathological pattern. Longitudinal follow-up of this and similar cohorts will be required to clarify the clinical trajectory and to determine whether tissue IgG4-rich status carries independent prognostic information.

Principal strengths of this study are the integration of tissue immunohistochemistry, BAL and peripheral blood flow cytometry, chest CT, and pulmonary function in a single consecutive clinical cohort, with a detailed characterization of RA disease activity and treatment, and the use of exploratory sensitivity and subgroup analyses to test the robustness of the principal observations. Several limitations must be acknowledged. First, the study is single-center and retrospective, with a modest overall sample size (n=28) and only six IgG4-rich cases; CIs are correspondingly wide and the multivariable analyses are exploratory. Second, all IgG4-rich patients were men and ever-smokers, creating quasi-complete separation that we addressed with Firth penalized logistic regression but that nevertheless prevents statistical dissection of the independent contributions of male sex and smoking. Accordingly, the Firth regression is presented as a sensitivity analysis rather than as evidence of an independent association. Third, although we used a hot-spot field selection approach and visual verification, formal inter-observer reproducibility of the IgG4/IgG ratio was not quantified in the present cohort and is acknowledged as a limitation; standardized, multi-observer reassessment is planned for a future cohort. Relatedly, the plasma-cell identity of the IgG4-positive cells was not confirmed by double or multiplex immunostaining with plasma-cell markers such as CD38, CD138, or IRF4. Although the IgG4-positive cells exhibited morphological features consistent with plasma cells, IgG4 immunostaining and morphology alone cannot definitively establish their cellular identity; we therefore use the term “IgG4-positive cells” and avoid attributing these findings specifically to plasma-cell infiltration. Fourth, chest CT findings were abstracted from clinical radiology reports rather than re-evaluated prospectively by blinded readers. The absence of standardized, blinded central CT review may have introduced classification variability, particularly for reported UIP pattern and emphysema severity; imaging findings are therefore presented descriptively rather than as primary outcomes. Fifth, TBLB samples are small and may not be representative of whole-lung pathology. Sixth, all participants had abnormal chest imaging that prompted BAL and TBLB, so the findings cannot be extrapolated to RA patients with normal lungs; ethically, no healthy-lung comparison group was feasible. Finally, multiple testing was not corrected for in the principal analyses, and the analyses are hypothesis-generating rather than confirmatory.

In this exploratory, single-center, retrospective cohort of RA patients with abnormal lung imaging, IgG4-positive cell infiltration (exploratory tissue IgG4/IgG ratio ≥10%) was observed in a small subset of patients, all of whom were male ever-smokers, and was accompanied by differences in the BAL immune-cell profile and co-occurring smoking-related structural changes on CT. Given the small sample size, the exploratory IgG4/IgG cutoff, the fact that only one patient met the conventional ≥40% threshold, and the unresolved confounding between male sex and smoking, these observations should be regarded as hypothesis-generating rather than as establishing a distinct RA-lung phenotype. Larger prospective, multicenter studies with standardized pathological and radiological assessment are required to determine whether IgG4-positive lung infiltration represents a reproducible RA-associated finding.

## Data Availability

The raw data supporting the conclusions of this article will be made available by the authors, on reasonable request and subject to institutional data-sharing and ethical-approval policies.

## References

[1] Lanzillotta M Mancuso G Della-Torre E . Advances in the diagnosis and management of IgG4 related disease. BMJ. (2020) 369:m1067. doi: 10.1136/bmj.m1067 32546500

[2] Umehara H Okazaki K Kawa S Tsuboi H Hirano K Ohara H . The 2020 revised comprehensive diagnostic (RCD) criteria for IgG4-RD. Mod Rheumatol. (2021) 31:529–33. doi: 10.1080/14397595.2020.1859710 33274670

[3] Wallace ZS Naden RP Chari S Choi HK Della-Torre E Dicaire J-F . The 2019 american college of rheumatology/european league against rheumatism classification criteria for igG4-related disease. Ann Rheum Dis. (2020) 79:77–87. doi: 10.1136/annrheumdis-2019-216561 31796497

[4] Ryu JH Sekiguchi H Yi ES . Pulmonary manifestations of immunoglobulin G4-related sclerosing disease. Eur Respir J. (2012) 39:180–6. doi: 10.1183/09031936.00025211 21719489

[5] Matsui S Yamamoto H Minamoto S Waseda Y Mishima M Kubo K . Proposed diagnostic criteria for IgG4-related respiratory disease. Respir Investig. (2016) 54:130–2. doi: 10.1016/j.resinv.2015.09.002 26879484

[6] Matteson EL Matucci-Cerinic M Kreuter M Buch MH Lin C-J Tony H-P . Patterns of interstitial lung disease and mortality in rheumatoid arthritis. Rheumatol (Oxford). (2017) 56:344–50. doi: 10.1093/rheumatology/kew391 27940586 PMC6251572

[7] Kronzer VL Huang W Dellaripa PF Doyle TJ Sparks JA . Lifestyle and clinical risk factors for incident rheumatoid arthritis-associated interstitial lung disease. J Rheumatol. (2021) 48:656–63. doi: 10.3899/jrheum.200863 33191286 PMC8096643

[8] Wallwork R Perugino CA Fu X Harkness T Zhang Y Choi HK . The association of smoking with immunoglobulin G4-related disease: a case-control study. Rheumatol (Oxford). (2021) 60:5310–7. doi: 10.1093/rheumatology/keab172 33751033 PMC8783539

[9] Tsuji Y Koga T Umeda M Sumiyoshi R Takatani A Igawa T . Identification of risk factors for elevated serum IgG4 levels in subjects in a large-scale health checkup cohort study. Front Immunol. (2023) 14:1124417. doi: 10.3389/fimmu.2023.1124417 36969256 PMC10031005

[10] Tsuge S Fujii H Tamai M Goto Y Hosoba R Yokota K . Factors related to elevated serum immunoglobulin G4 (IgG4) levels in a Japanese general population. Arthritis Res Ther. (2024) 26:156. doi: 10.1186/s13075-024-03390-x 39242517 PMC11378454

[11] Mattoo H Mahajan VS Maehara T Deshpande V Della-Torre E Wallace ZS . Clonal expansion of CD4+ cytotoxic T lymphocytes in patients with IgG4-related disease. J Allergy Clin Immunol. (2016) 138:825–38. doi: 10.1016/j.jaci.2015.12.1330 26971690 PMC5014627

[12] Wallace ZS Mattoo H Carruthers M Mahajan VS Della-Torre E Deshpande V . Plasmablasts as a biomarker for IgG4-related disease, independent of serum IgG4 concentrations. Ann Rheum Dis. (2015) 74:190–5. doi: 10.1136/annrheumdis-2014-205233 24817416 PMC4656194

[13] Klareskog L Stolt P Lundberg K Källberg H Bengtsson C Grunewald J . A new model for an etiology of rheumatoid arthritis: smoking may trigger HLA-DR (shared epitope)-restricted immune reactions to autoantigens modified by citrullination. Arthritis Rheum. (2006) 54:38–46. doi: 10.1002/art.21575 16385494

[14] Aletaha D Neogi T Silman AJ Funovits J Felson DT Bingham CO . 2010 Rheumatoid arthritis classification criteria: an American College of Rheumatology/European League Against Rheumatism collaborative initiative. Ann Rheum Dis. (2010) 69:1580–8. doi: 10.1136/ard.2010.138461 20699241

[15] von Elm E Altman DG Egger M Pocock SJ Gøtzsche PC Vandenbroucke JP . The Strengthening the Reporting of Observational Studies in Epidemiology (STROBE) statement: guidelines for reporting observational studies. Lancet. (2007) 370:1453–7. doi: 10.1016/S0140-6736(07)61602-X 18064739

[16] Deshpande V Zen Y Chan JK Yi EE Sato Y Yoshino T . Consensus statement on the pathology of IgG4-related disease. Mod Pathol. (2012) 25:1181–92. doi: 10.1038/modpathol.2012.72 22596100

[17] Firth D . Bias reduction of maximum likelihood estimates. Biometrika. (1993) 80:27–38. doi: 10.1093/biomet/80.1.27

[18] Heinze G Schemper M . A solution to the problem of separation in logistic regression. Stat Med. (2002) 21:2409–19. doi: 10.1002/sim.1047 12210625

[19] Virtanen P Gommers R Oliphant TE Haberland M Reddy T Cournapeau D . SciPy 1.0: fundamental algorithms for scientific computing in Python. Nat Methods. (2020) 17:261–72. doi: 10.1038/s41592-019-0686-2 32015543 PMC7056644

[20] van der Walt S Schönberger JL Nunez-Iglesias J Boulogne F Warner JD Yager N . scikit-image: image processing in Python. PeerJ. (2014) 2:e453. doi: 10.7717/peerj.453 25024921 PMC4081273

[21] Carruthers MN Khosroshahi A Augustin T Deshpande V Stone JH . The diagnostic utility of serum IgG4 concentrations in IgG4-related disease. Ann Rheum Dis. (2015) 74:14–8. doi: 10.1136/annrheumdis-2013-204907 24651618

[22] Perugino CA Stone JH . IgG4-related disease: an update on pathophysiology and implications for clinical care. Nat Rev Rheumatol. (2020) 16:702–14. doi: 10.1038/s41584-020-0500-7 32939060

