# Supplementary material for: Smoking-associated IgG4-positive cell infiltration in rheumatoid arthritis lung: an exploratory cross-sectional study
Source: Front Immunol. 2026 Aug 14;17:1873384. doi: 10.3389/fimmu.2026.1873384 (PMC13522123; doi:10.3389/fimmu.2026.1873384)

Supplementary File 1. STROBE checklist for cross-sectional studies

This checklist follows the STROBE statement (von Elm E, et al. Lancet 2007;370:1453–7) for cross-sectional observational studies. Page numbers refer to the main manuscript (Manuscript_RMDOpen_v9_main.docx).

Item No.	Recommendation	Description in this manuscript	Page
1a	Title and abstract — indicate study design	Cross-sectional design indicated in Materials and Methods 2.1; design also reflected in the Abstract opening sentence.	Title page; Abstract
1b	Title and abstract — informative summary	Single-paragraph structured abstract per Frontiers in Immunology format, sequentially covering background, methods, results and conclusion.	Abstract
2	Background/ rationale — explain scientific background	Introduction synthesizes IgG4-RD epidemiology and pathology criteria, smoking and IgG4-RD/RA-ILD literature, and our prior NaIS cohort findings, before stating the rationale for tissue-level investigation.	Introduction
3	Objectives — state specific objectives	Last paragraph of Introduction explicitly states that the aim is to examine whether IgG4-rich plasma-cell infiltration is detectable in RA lung tissue and is associated with smoking-related lung changes; we explicitly do not aim to diagnose IgG4-RD.	Introduction
4	Study design — present key elements early	Single-center, retrospective, cross-sectional study at Nagasaki University Hospital; reported with the STROBE statement.	Materials and Methods 2.1
5	Setting — describe setting, locations, dates	Nagasaki University Hospital, June 2019 – October 2021.	Materials and Methods 2.1
6a	Participants — eligibility, sources, methods of selection	Consecutive RA patients (ACR/EULAR 2010) undergoing BAL and TBLB for evaluation of abnormal pulmonary shadows.	Materials and Methods 2.1
7	Variables — define all outcomes, exposures, confounders	Outcome: tissue IgG4/IgG ratio (continuous and ≥10% threshold); exposures: sex, smoking, ACPA, UIP pattern; potential confounders: RA activity, treatment, comorbidities, age.	Materials and Methods 2.2, 2.4
8	Data sources/measurement	Tissue immunohistochemistry (IgG, IgG4); BAL and PBMC flow cytometry (full antibody panel); chest CT (radiology reports); pulmonary function tests; clinical records for RA activity and treatment.	Materials and Methods 2.2–2.4
9	Bias — describe efforts to address potential sources of bias	Hot-spot field selection for IgG/IgG4 quantification with manual visual verification of three morphological criteria; explicit exclusion of dust-laden macrophages and edge artifacts; sensitivity analyses across thresholds; subgroup analyses to address sex/smoking/ILD confounding.	Materials and Methods 2.2, 2.5
10	Study size — explain how sample	Convenience sample; all eligible patients with available tissue specimens during the recruitment window were included (n=28).	Materials and

Item No.	Recommendation	Description in this manuscript	Page
	size was arrived at		Methods 2.1
11	Quantitative variables — describe handling	Continuous variables analyzed as median [Q1, Q3]; tissue IgG4 also dichotomized at ≥10% (exploratory); censored ACPA/IgE values handled by rank-based methods.	Materials and Methods 2.5
12a	Statistical methods — describe all statistical methods	Mann–Whitney U; Fisher exact; Spearman correlation; Firth penalized logistic regression for multivariable model.	Materials and Methods 2.5
12b	Subgroup and sensitivity analyses	Pre-specified sensitivity analyses at thresholds 5/10/20/40%; subgroup analyses among men only, ever-smokers only and ILD-positive only.	Materials and Methods 2.5; Results 3.6, 3.7
12c	Missing data	Continuous variables analyzed only when measured; sample sizes per variable reported in tables; no imputation performed.	Tables 2, 3; Materials and Methods 2.5
13	Participants — number at each stage	28 of consecutive eligible patients with available tissue specimens were included; flow described in Materials and Methods 2.1.	Materials and Methods 2.1
14	Descriptive data — characteristics of study participants	Table 1 presents demographic, smoking, serological and treatment characteristics by tissue IgG4 group.	Table 1
15	Outcome data — report numbers of outcome events	Tissue IgG4-rich group n=6; IgG4-low group n=22; only one patient met the IgG4-RD pathology threshold ≥40%.	Results 3.1
16	Main results — give unadjusted and (where appropriate) adjusted estimates	Descriptive comparisons in Tables 1–3; exploratory Firth multivariable analysis in Table 4 with explicit caveat that primary inference is descriptive.	Tables 1–4
17	Other analyses — subgroup and sensitivity analyses	Sensitivity (Supplementary Table S1) and subgroup analyses (Supplementary Tables S2–S4) reported in supplementary material; continuous Spearman correlations (Table S5); peripheral blood flow cytometry (Table S6).	Supplementary material
18	Key results	Summarized in Discussion (paragraph 1).	Discussion
19	Limitations	Sample size, quasi-complete separation, single-center, retrospective, hot-spot pathology with no formal inter-observer assessment, CT abstracted from clinical reports, no healthy control, no multiple-testing correction.	Discussion (limitations paragraph)
20	Interpretation	Cautious causal language; explicit acknowledgment that we cannot determine whether tissue IgG4-rich infiltration represents preclinical	Discussion

Item No.	Recommendation	Description in this manuscript	Page
		IgG4-RD, a nonspecific response or a distinct RA-lung phenotype.	
21	Generalizability	Findings cannot be extrapolated to RA patients with normal lungs, as participants were selected on abnormal CT findings.	Discussion (limitations paragraph)
22	Funding	Sources of funding stated in dedicated section.	Funding

Table S1. Sensitivity analysis: associations of tissue IgG4 status with sex, smoking and ACPA across cutoff thresholds (5%, 10%, 20%, 40%)

Cutoff	n IgG4-pos	n IgG4-neg	Male, pos vs neg	Ever-smoker, pos vs neg	Smoking p	Smoking OR	ACPA p
≥5%	7	21	6/7 (86%) vs 3/21 (14%)	6/7 (86%) vs 7/21 (33%)	0.029	12.00	0.038
≥10%	6	22	6/6 (100%) vs 3/22 (14%)	6/6 (100%) vs 7/22 (32%)	0.005	∞	0.015
≥20%	5	23	5/5 (100%) vs 4/23 (17%)	5/5 (100%) vs 8/23 (35%)	0.013	∞	0.058
≥40%	1	27	1/1 (100%) vs 8/27 (30%)	1/1 (100%) vs 12/27 (44%)	0.464	∞	-

Sensitivity analysis at four thresholds for tissue IgG4 positivity. The principal between-group analysis used the ≥10% threshold (exploratory operational definition of IgG4-rich infiltration). The IgG4-RD pathology threshold of ≥40% yielded only one positive case in this cohort. Fisher exact test for proportions and Mann–Whitney U test for ACPA. OR with cell zeros was computed using Haldane–Anscombe correction (+0.5 to all cells).

Table S2. Subgroup analysis among men only (n=9)

Tissue IgG4-rich (≥10%) n=6 versus IgG4-low n=3.

Variable	IgG4-low	IgG4-rich	p
Age (years)	68.00 [62.50, 74.50]	67.50 [64.75, 71.00]	0.896
Pack-years (ever-smokers only)	1500.00 [1350.00, 1530.00]	900.00 [780.00, 975.00]	0.037
ACPA (U/mL)	0.60 [0.60, 35.80]	343.05 [303.32, 461.75]	0.027
Serum IgG4 (mg/dL)	76.30 [70.25, 256.65]	83.65 [65.45, 98.70]	0.905
CRP (mg/dL)	2.22 [1.52, 3.08]	0.78 [0.57, 2.70]	0.548
DAS28-CRP	3.31 [3.24, 3.41]	3.80 [3.44, 4.18]	0.381
IgG4+ cells/HPF	0.00 [0.00, 0.00]	12.00 [4.00, 53.00]	0.025
BAL CD4/CD8 ratio	1.30 [1.00, 1.90]	1.05 [0.77, 1.18]	0.437
BAL CD22+ B cells (%)	1.60 [1.00, 2.70]	3.35 [2.67, 4.40]	0.300
BAL CD8+ (%)	35.00 [30.70, 45.20]	44.65 [42.88, 45.97]	0.714
BAL Treg, CD127-CD25+ (% of BAL CD4+)	23.60 [17.15, 23.80]	21.80 [12.74, 33.42]	0.905
Baseline %FEV1.0	103.60 [99.90, 108.70]	73.25 [64.72, 91.67]	0.048
ILD on chest CT	2/3 (67%)	6/6 (100%)	0.333
UIP pattern on chest CT	1/3 (33%)	1/6 (17%)	1.000
Male sex	3/3 (100%)	6/6 (100%)	1.000
Ever-smoker	3/3 (100%)	6/6 (100%)	1.000

Mann–Whitney U for continuous variables and Fisher exact test for categorical variables. Yellow shading: $p<0.05$; orange shading: $0.05 \le p < 0.10$.

Table S3. Subgroup analysis among ever-smokers only (n=13)

Tissue IgG4-rich (≥10%) n=6 versus IgG4-low n=7.

Variable	IgG4-low	IgG4-rich	p
Age (years)	61.00 [58.50, 72.50]	67.50 [64.75, 71.00]	0.616
Pack-years (ever-smokers only)	420.00 [157.50, 1350.00]	900.00 [780.00, 975.00]	0.667
ACPA (U/mL)	71.00 [13.25, 284.95]	343.05 [303.32, 461.75]	0.052
Serum IgG4 (mg/dL)	64.20 [56.45, 103.15]	83.65 [65.45, 98.70]	0.628
CRP (mg/dL)	0.83 [0.41, 3.08]	0.78 [0.57, 2.70]	1.000
DAS28-CRP	3.31 [2.40, 3.51]	3.80 [3.44, 4.18]	0.181
IgG4+ cells/HPF	0.00 [0.00, 0.00]	12.00 [4.00, 53.00]	0.002
BAL CD4/CD8 ratio	1.80 [1.15, 2.30]	1.05 [0.77, 1.18]	0.099
BAL CD22+ B cells (%)	2.50 [1.30, 2.90]	3.35 [2.67, 4.40]	0.151
BAL CD8+ (%)	35.00 [28.15, 39.90]	44.65 [42.88, 45.97]	0.181
BAL Treg, CD127-CD25+ (% of BAL CD4+)	23.60 [12.70, 27.25]	21.80 [12.74, 33.42]	0.945
Baseline %FEV1.0	103.60 [95.40, 113.65]	73.25 [64.72, 91.67]	0.022
ILD on chest CT	3/7 (43%)	6/6 (100%)	0.070
UIP pattern on chest CT	1/7 (14%)	1/6 (17%)	1.000
Male sex	3/7 (43%)	6/6 (100%)	0.070
Ever-smoker	7/7 (100%)	6/6 (100%)	1.000

Restricting the cohort to ever-smokers attenuates but does not eliminate the principal differences. IgG4+ cells/HPF, baseline %FEV1.0 and ACPA all retain trend or significant differences.

Table S4. Subgroup analysis among patients with CT-defined ILD (n=14)

Tissue IgG4-rich (≥10%) n=6 versus IgG4-low n=8, all with ILD on chest CT.

Variable	IgG4-low	IgG4-rich	p
Age (years)	68.00 [67.75, 72.75]	67.50 [64.75, 71.00]	0.513
Pack-years (ever-smokers only)	1200.00 [810.00, 1350.00]	900.00 [780.00, 975.00]	0.603
ACPA (U/mL)	159.20 [67.33, 299.60]	343.05 [303.32, 461.75]	0.136
Serum IgG4 (mg/dL)	72.85 [61.90, 101.20]	83.65 [65.45, 98.70]	0.852
CRP (mg/dL)	3.04 [0.79, 4.04]	0.78 [0.57, 2.70]	0.491
DAS28-CRP	4.08 [3.28, 4.44]	3.80 [3.44, 4.18]	1.000
IgG4+ cells/HPF	0.00 [0.00, 1.50]	12.00 [4.00, 53.00]	0.019
BAL CD4/CD8 ratio	2.50 [1.15, 4.95]	1.05 [0.77, 1.18]	0.121
BAL CD22+ B cells (%)	1.40 [0.73, 2.62]	3.35 [2.67, 4.40]	0.053
BAL CD8+ (%)	27.30 [16.48, 40.10]	44.65 [42.88, 45.97]	0.181
BAL Treg, CD127-CD25+ (% of BAL CD4+)	12.10 [3.46, 23.70]	21.80 [12.74, 33.42]	0.345
Baseline %FEV1.0	108.55 [97.03, 118.30]	73.25 [64.72, 91.67]	0.013
ILD on chest CT	8/8 (100%)	6/6 (100%)	1.000
UIP pattern on chest CT	2/8 (25%)	1/6 (17%)	1.000
Male sex	2/8 (25%)	6/6 (100%)	0.010
Ever-smoker	3/8 (38%)	6/6 (100%)	0.031

Within ILD-positive patients, IgG4-rich status is still associated with male sex, ever-smoking, higher IgG4+ cell counts and lower baseline %FEV1.0, suggesting that tissue IgG4-rich infiltration is not solely a marker of ILD.

Table S5. Spearman rank correlations between continuous tissue IgG4 measures and other variables (n=28)

Tissue measure (x) vs variable (y)	n	ρ	p-value	Significance
IgG4/IgG ratio (%) vs BAL CD4/CD8	27	-0.283	0.153	
IgG4/IgG ratio (%) vs BAL CD22 (%)	27	+0.319	0.105	
IgG4/IgG ratio (%) vs BAL CD8 (%)	27	+0.259	0.192	
IgG4/IgG ratio (%) vs BAL Treg (%)	27	+0.105	0.604	
IgG4/IgG ratio (%) vs Pack-years (smokers)	13	-0.004	0.989	
IgG4/IgG ratio (%) vs ACPA	28	+0.499	0.007	*
IgG4/IgG ratio (%) vs Serum IgG4	28	+0.244	0.211	
IgG4/IgG ratio (%) vs Baseline %FEV1.0	27	-0.390	0.044	*
IgG4+ cells/HPF vs BAL CD4/CD8	27	-0.133	0.508	
IgG4+ cells/HPF vs BAL CD22 (%)	27	+0.197	0.325	
IgG4+ cells/HPF vs BAL CD8 (%)	27	+0.126	0.533	
IgG4+ cells/HPF vs BAL Treg (%)	27	+0.025	0.901	
IgG4+ cells/HPF vs Pack-years (smokers)	13	+0.089	0.774	
IgG4+ cells/HPF vs ACPA	28	+0.443	0.018	*
IgG4+ cells/HPF vs Serum IgG4	28	+0.253	0.194	
IgG4+ cells/HPF vs Baseline %FEV1.0	27	-0.333	0.090	†

*Spearman rank correlation. * $p<0.05$, † $0.05 \le p < 0.10$. Continuous tissue measures: tissue IgG4/IgG ratio (%), and IgG4+ cells per high-power field (HPF). Pack-years analyses are restricted to ever-smokers (n=13).*

Table S6. Peripheral blood flow-cytometric findings, stratified by tissue IgG4/IgG ratio

Variable	n (IgG4-rich)	IgG4-rich [Q1, Q3]	n (IgG4-low)	IgG4-low [Q1, Q3]	p
PB WBC (×10³/μL)	6	6.35 [4.78, 7.33]	21	6.60 [5.10, 8.50]	0.502
PB lymphocytes (×10³/μL)	6	1.39 [1.34, 1.53]	21	1.39 [1.18, 1.71]	0.930
PB monocytes (×10³/μL)	6	0.33 [0.30, 0.34]	21	0.37 [0.26, 0.44]	0.321
PB eosinophils (×10³/μL)	6	0.17 [0.15, 0.22]	21	0.12 [0.09, 0.24]	0.220
PB basophils (×10³/μL)	6	0.03 [0.01, 0.03]	21	0.03 [0.02, 0.05]	0.246
PB neutrophils (×10³/μL)	6	4.75 [3.18, 5.16]	21	4.30 [3.40, 5.83]	0.683
PB Treg, CD127-CD25+ (%)	6	6.21 [4.58, 8.41]	20	4.97 [4.13, 5.74]	0.494
PB Th, CCR6+CXCR3- (%)	6	13.65 [11.25, 15.82]	19	14.80 [11.80, 19.75]	0.656
PB Th17, CCR6+CXCR3+ (%)	6	9.65 [9.36, 11.83]	19	16.80 [7.24, 20.55]	0.780
PB Th2, CCR6-CXCR3- (%)	6	45.80 [38.15, 52.85]	19	32.10 [19.25, 55.55]	0.514
PB Th1, CCR6-CXCR3+ (%)	6	30.50 [23.82, 35.45]	19	28.10 [19.80, 42.95]	1.000
PB macrophages (CD14+CD16-, %)	6	22.75 [10.81, 38.62]	20	40.80 [22.65, 69.22]	0.139
PB activated macrophages (CD86+CD14+, %)	6	20.58 [8.09, 48.57]	20	26.55 [15.40, 36.38]	0.929
PB neutrophils (CD66b+CD16+, %)	2	78.85 [78.78, 78.92]	5	73.90 [63.00, 76.20]	0.381
PB neutrophils (CD66b+CD206-, %)	6	83.60 [75.62, 85.88]	18	53.05 [39.27, 73.47]	0.104

Mann–Whitney U test. None of the peripheral blood T-helper subsets reached statistical significance, in contrast with the BAL fluid panel (Table 2 of the main manuscript). Serum IgG4, RF, anti-CCP antibody, ESR and CRP are reported in Table 1 of the main manuscript.

PB, peripheral blood; PBMC, peripheral blood mononuclear cell; BAL, bronchoalveolar lavage.

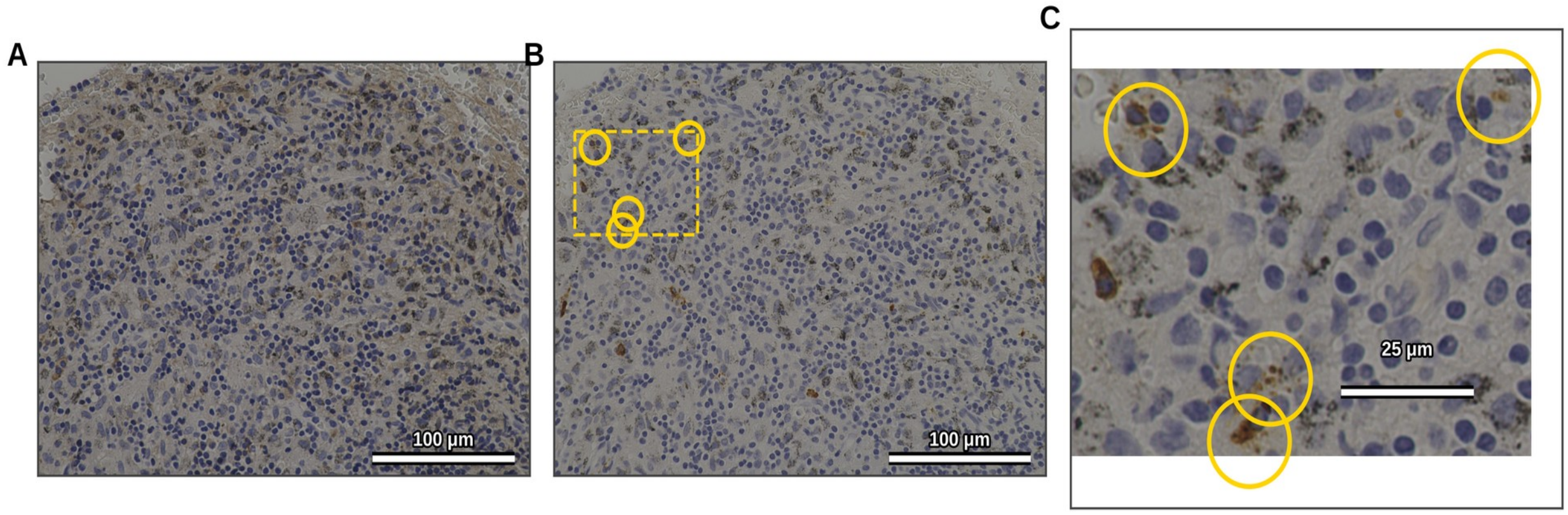

Supplementary Figure S1. Representative immunohistochemistry of an IgG4-rich case (patient KY-8; male, ever-smoker; tissue IgG4/IgG ratio = 26.0%; 80 IgG4-positive and 308 IgG-positive cells per high-power field). **(A)** IgG immunostaining showing a robust IgG-positive cell infiltrate at ×400. **(B)** Matched IgG4 immunostaining of the same tissue area at ×400. Yellow circles indicate representative IgG4-positive cells satisfying the morphological criteria (clearly visible nuclear-negative area, homogeneous DAB-positive cytoplasm, recognition as an isolated, independent cell). The dashed yellow rectangle marks the region shown at higher magnification in panel C. **(C)** High-magnification view of the boxed area in panel B; the same IgG4-positive cells are again outlined. Scale bars indicate 100 µm in panels A and B and 25 µm in panel C. Original images were captured at 1920 × 1440 pixels; pixel-to-micrometre conversion is approximate.

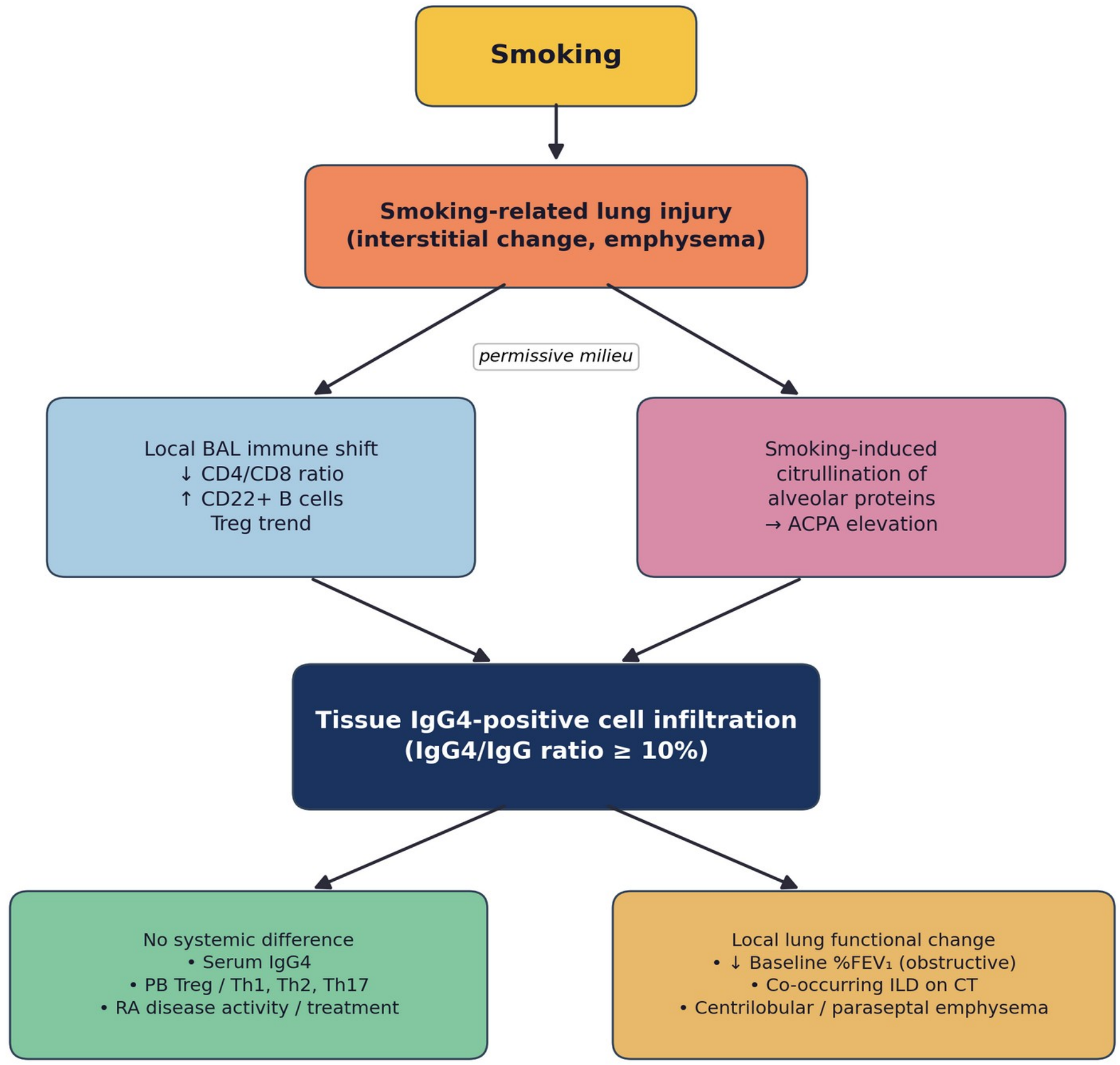

Supplementary Figure S2. Conceptual summary of smoking-related local IgG4-positive cell infiltration in rheumatoid arthritis (RA) lung. Smoking is hypothesized to act through two parallel routes — a local immune shift in bronchoalveolar lavage (BAL) fluid (lower CD4/CD8 ratio, expanded CD22+ B cells, and a trend toward increased regulatory T cells) and citrullination of alveolar proteins driving anti-citrullinated peptide antibody (ACPA) elevation — that converge on tissue IgG4-positive cell infiltration (defined here as a tissue IgG4/IgG ratio ≥10%). This local pattern is not accompanied by differences in serum IgG4, peripheral blood T-helper subsets, or RA disease activity and treatment, but it is paralleled by reduced baseline percent-predicted FEV1 and by co-occurring interstitial lung disease and emphysema on chest computed tomography. Solid arrows indicate the proposed conceptual flow; the model is hypothesis-generating.

Supplementary Figure S3. Gating strategy for BAL fluid and peripheral blood T-cell subsets. Representative sample analyzed in FlowJo. Lymphocytes were gated on FSC-A/SSC-A, then CD4+ T cells; within CD4+ cells, regulatory T cells were defined as CD25+CD127− (Treg gate) and T-helper subsets by CCR6/CXCR3 (Th1: CCR6−CXCR3+; Th2: CCR6−CXCR3−; Th17: CCR6+CXCR3+; Q1 = CCR6+CXCR3−). Naïve/memory subsets are shown by CCR7/CD45RA. Quadrant values are percentages of the parent gate. Top row, peripheral blood; bottom row, BAL fluid.

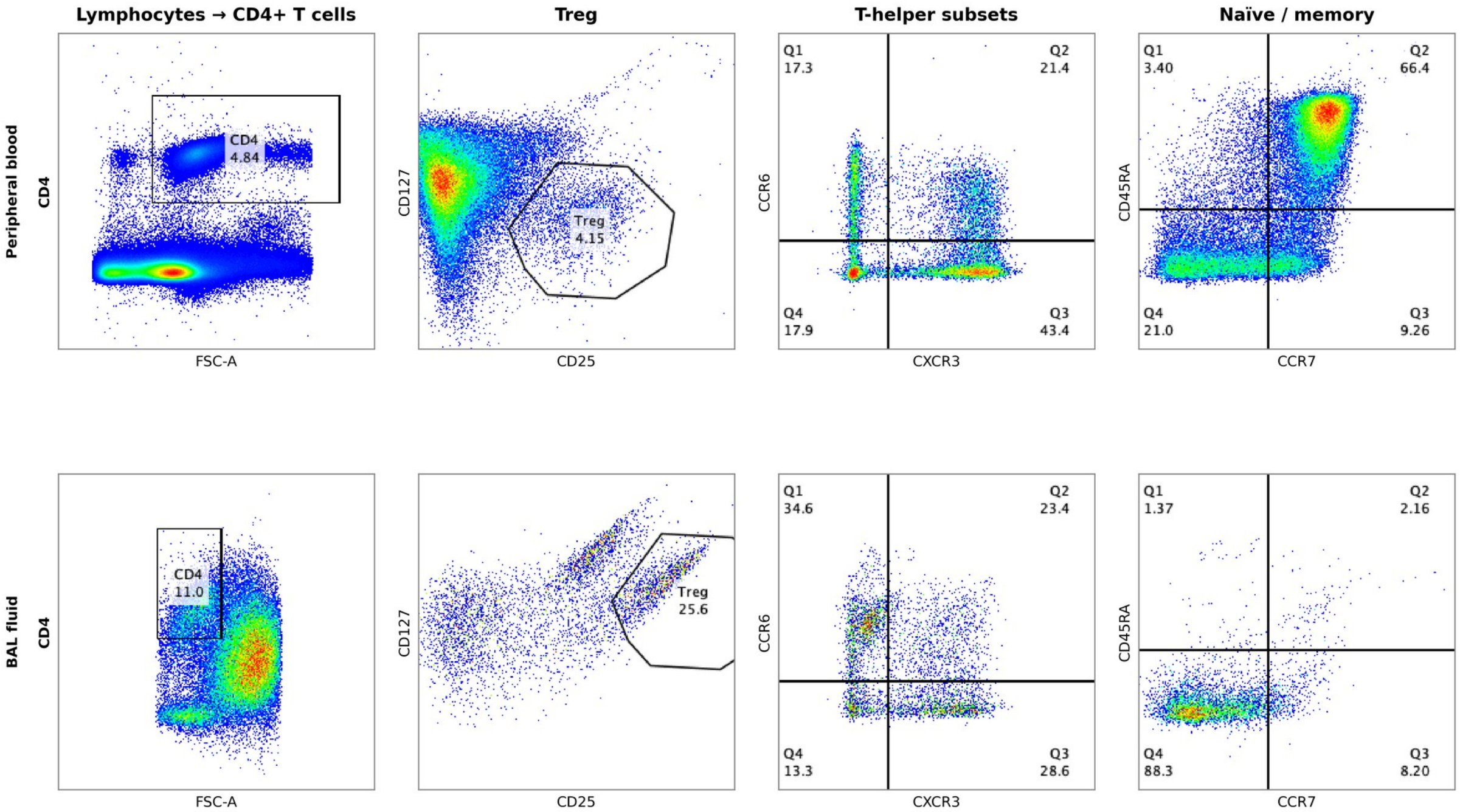

Supplement: Supplementary file 1 [file DataSheet1.pdf]
